# (*R*)-(+)-2-{[(3-Methyl-4-nitro­pyridin-2-yl)meth­yl]sulfin­yl}-1*H*-benzimidazole^1^

**DOI:** 10.1107/S1600536811029990

**Published:** 2011-07-30

**Authors:** Manne Naga Raju, Neelam Uday Kumar, Naveenkumar Kolla, Rakeshwar Bandichhor, Peddy Vishweshwar

**Affiliations:** a Research and Development, API-PDT-03, Integrated Product Development (IPD), Innovation Plaza, Dr Reddy’s Laboratories Ltd, Bachupally, Qutubullapur, Hyderabad 500 072, India; b Centre of Excellence in Polymorphism and Particle Engineering, Integrated Product Development (IPD), Innovation Plaza, Dr Reddy’s Laboratories Ltd, Bachupally, Qutubullapur, Hyderabad 500 072, India

## Abstract

The title compound, C_14_H_12_N_4_O_3_S, is an inter­mediate of Dexlansoprazole, a proton pump inhibitor (PPI) mainly developed for anti-ulcer activity. The absolute configuration of the title compound was determined as *R*. The crystal structure reveals that the mol­ecules form chains along the *b* axis through N—H⋯N and C—H⋯O hydrogen-bonded dimers. These chains are connected *via* weak C—H⋯O hydrogen bonds.

## Related literature

For the synthesis of the title compound, see: Kumar *et al.* (2009 ▶). For background to this class of anti-ulcer drugs, see: Arimori *et al.* (1998 ▶); Masa *et al.* (2001 ▶). For a related structure, see: Fujishima *et al.* (2002 ▶).
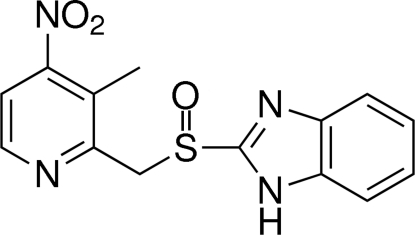


## Experimental

### 

#### Crystal data

C_14_H_12_N_4_O_3_S
*M* *_r_* = 316.33
Monoclinic, 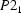

*a* = 7.7422 (13) Å
*b* = 11.0505 (15) Å
*c* = 8.2318 (13) Å
β = 103.697 (7)°
*V* = 684.24 (18) Å^3^
*Z* = 2
Mo *K*α radiation
μ = 0.26 mm^−1^
*T* = 298 K
0.22 × 0.20 × 0.18 mm

#### Data collection

Rigaku Mercury diffractometer
Absorption correction: multi-scan (*REQAB*; Jacobson, 1998 ▶) *T*_min_ = 0.942, *T*_max_ = 0.950
7636 measured reflections
2752 independent reflections
2601 reflections with *F* ^2^ > 2σ(*F* ^2^)
*R*_int_ = 0.025

#### Refinement

*R*[*F* ^2^ > 2σ(*F* ^2^)] = 0.037
*wR*(*F* ^2^) = 0.038
*S* = 1.25
2752 reflections
215 parameters
H atoms treated by a mixture of independent and constrained refinement
Δρ_max_ = 0.48 e Å^−3^
Δρ_min_ = −0.37 e Å^−3^
Absolute structure: Flack (1983 ▶), with 1292 Friedel pairs
Flack parameter: −0.02 (4)

### 

Data collection: *CrystalClear* (Rigaku, 2005 ▶); cell refinement: *CrystalClear*; data reduction: *CrystalStructure* (Molecular Structure Corporation & Rigaku, 2006 ▶); program(s) used to solve structure: *SIR2004* (Burla *et al.* 2005 ▶); program(s) used to refine structure: *CRYSTALS* (Betteridge *et al.* 2003 ▶); molecular graphics: *X-SEED* (Barbour, 2001 ▶); software used to prepare material for publication: *CrystalStructure*.

## Supplementary Material

Crystal structure: contains datablock(s) global, I. DOI: 10.1107/S1600536811029990/gw2104sup1.cif

Structure factors: contains datablock(s) I. DOI: 10.1107/S1600536811029990/gw2104Isup3.hkl

Supplementary material file. DOI: 10.1107/S1600536811029990/gw2104Isup3.cml

Additional supplementary materials:  crystallographic information; 3D view; checkCIF report

## Figures and Tables

**Table 1:** Hydrogen-bond geometry (Å, °)

*D*—H⋯*A*	*D*—H	H⋯*A*	*D*⋯*A*	*D*—H⋯*A*
N1—H1⋯N2^i^	0.881 (17)	2.553 (18)	3.425 (2)	170.5 (13)
C2—H2⋯O1^ii^	0.95	2.33	3.251 (2)	164
C12—H12⋯O2^iii^	0.95	2.55	3.164 (2)	122

Symmetry codes: (i) 
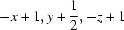
; (ii) 
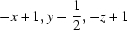
; (iii) 
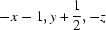
.

**Fig. 1.**
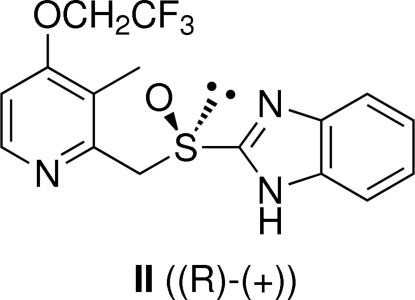
Schematic diagram of Dexlansoprazole (II) ((R)-(+)).

**Fig. 2.**
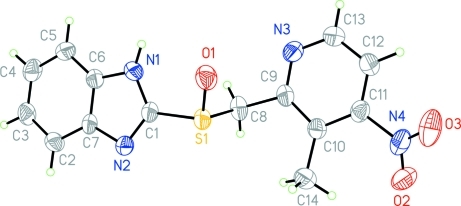
Molecular structure of (I), showing the atom numbering scheme. The displacement ellipsoids are drawn at the 50% probability level. H atoms are shown by small circles of arbitrary radii.

**Fig. 3.**
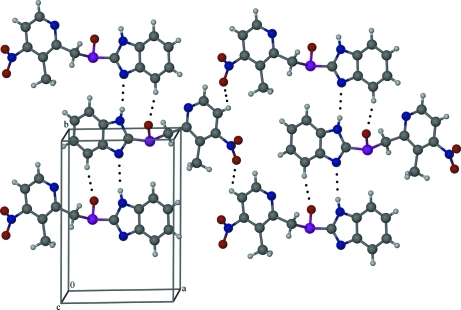
Crystal packing of (I).

## supplementary crystallographic information

### Comment

Dexlansoprazole II ((*R*)-(+)) (Fig. 1), (*R*)-enantiomer of Lansoprazole, is a proton pump inhibitor (PPI) mainly developed for anti-ulcer activity by TAP Pharmaceuticals Ltd., employing new modified-release technology (Arimori *et al.* 1998; Masa *et al.* 2001). Dexlansoprazole II ((*R*)-(+)) was first approved by United States Food and Drug Administration (US-FDA) in the form of 30 and 60 mg capsules for the management of patients with erosive oesophagitis and non-erosive reflux disease (GERD or GORD), under the brand name of DEXILANT.

An alternative and large-scale synthetic method for II ((*R*)-(+)) was developed in our laboratory by employing asymmetric oxidation conditions on prochiral nitrosulfide intermediate to yield enantiomerically enriched nitro sulphoxide derivative of the title compound I ((*R*)-(+)) as first stage intermediate (>90% ee) (Kumar *et al.* 2009). Titanium derived chiral complex (2.2:1.1:0.6 ratio of Titanium (IV)-*i*-propoxide:(+)-Diethyl *L*-tartrate:Water) was used in the reaction to induce the chirality. The enantiomerically enriched title compound I ((*R*)-(+)) as a resultant was subjected to acetone mediated crystallization to yield enantiopure I ((R)-(+)) (>97% ee) which on treatment with potassium salt of 2,2,2-triflouroethanol in dimethylformamide (DMF) yielded Dexlansoprazole II ((*R*)-(+)) with ICH quality having >99.8% ee.

The structure and stereochemistry of Dexlansoprazole II ((*R*)-(+)) was well established in the literature with various spectroscopic and single-crystal X-ray diffraction (Fujishima *et al.* 2002). Herein we have determined the absolute configuration of the title compound I as `*R*' by anomalous dispersion (Fig. 2). The Flack parameter value, -0.02 (4) for the assigned absolute configuration, suggest that it is correct with high accuracy. The title compound is enantipure sulphoxide containing substituents of benzimidazole and 2-(3-methyl-4-nitro-pyridin-2-yl) methane moieties with dextro (*d*)- optical configuration. The crystal structure reveals that title molecules are forming chains along the *b* axis through N_1_—H···N_2_ and C_2_—H···O_1_ hydrogen-bonded dimers. Such chains are connected *via* weak C_12_—H···O_2_ hydrogen bonds (Fig. 3).

### Experimental

A mixture of enantiomerically enriched title compound I ((*R*)-(+)) (12 g, 0.038 mol) and acetone (264 ml) were heated to 45–50 °C until clear solution obtained. The resulting clear solution was cooled to -5 to 0 °C and stirred for 1.0–1.5 h. The precipitated I (*RS*)-(±) was filtered and the filtrate was evaporated under vacuum at below 45 °C to obtain thick residue of the title compound I ((*R*)-(+)). The resulting thick residue of the title compound I ((*R*)-(+)) was dissolved in dichloromethane and kept for slow solvent evaporation to grow single crystals.

### Refinement

The C-bound H atoms were geometrically placed (C—H = 0.95 Å) and refined as riding with *U_iso_*(H) = 1.2*Ueq*(parent atom).

### Crystal data

**Table:**

C_14_H_12_N_4_O_3_S	*F*(000) = 328.00
*M**_r_* = 316.33	*D*_x_ = 1.535 Mg m^−^^3^
Monoclinic, *P*2_1_	Mo *K*α radiation, λ = 0.71070 Å
Hall symbol: P 2yb	Cell parameters from 4183 reflections
*a* = 7.7422 (13) Å	θ = 1.8–27.5°
*b* = 11.0505 (15) Å	µ = 0.26 mm^−^^1^
*c* = 8.2318 (13) Å	*T* = 298 K
β = 103.697 (7)°	Prism, colourless
*V* = 684.24 (18) Å^3^	0.22 × 0.20 × 0.18 mm
*Z* = 2	

### Data collection

**Table:**

Rigaku Mercury diffractometer	2601 reflections with *F*^2^ > 2σ(*F*^2^)
Detector resolution: 7.31 pixels mm^-1^	*R*_int_ = 0.025
ω scans	θ_max_ = 27.5°
Absorption correction: multi-scan (REQAB; Jacobson, 1998)	*h* = −10→10
*T*_min_ = 0.942, *T*_max_ = 0.950	*k* = −13→13
7636 measured reflections	*l* = −7→10
2752 independent reflections	

### Refinement

**Table:**

Refinement on *F*^2^	*w* = 1/[σ^2^(*F*_o_^2^) + (0.1*P*)^2^] where *P* = (*F*_o_^2^ + 2*F*_c_^2^)/3
*R*[*F*^2^ > 2σ(*F*^2^)] = 0.037	(Δ/σ)_max_ < 0.001
*wR*(*F*^2^) = 0.038	Δρ_max_ = 0.48 e Å^−^^3^
*S* = 1.25	Δρ_min_ = −0.37 e Å^−^^3^
2752 reflections	Absolute structure: Flack (1983), with 1292 Friedel pairs
215 parameters	Flack parameter: −0.02 (4)
H atoms treated by a mixture of independent and constrained refinement	

### Special details

**Table:**

Geometry. Bond distances, angles *etc*. have been calculated using the rounded fractional coordinates. All su's are estimated from the variances of the (full) variance-covariance matrix. The cell e.s.d.'s are taken into account in the estimation of distances, angles and torsion angles
Refinement. Refinement was performed using all reflections. The weighted *R*-factor (*wR*) and goodness of fit (*S*) are based on *F*^2^. *R*-factor (gt) are based on *F*. The threshold expression of *F*^2^ > 2.0 σ(*F*^2^) is used only for calculating *R*-factor (gt).

### Fractional atomic coordinates and isotropic or equivalent isotropic displacement parameters (Å^2^)

**Table:**

	*x*	*y*	*z*	*U*_iso_*/*U*_eq_	
S1	0.23549 (4)	0.46611 (4)	0.34638 (4)	0.0355 (1)	
O1	0.24188 (14)	0.57292 (10)	0.23887 (14)	0.0447 (3)	
O2	−0.55199 (16)	0.35772 (11)	0.11414 (16)	0.0604 (4)	
O3	−0.57873 (16)	0.49507 (15)	−0.07757 (16)	0.0692 (5)	
N1	0.51652 (18)	0.57978 (12)	0.55851 (18)	0.0374 (4)	
N2	0.52568 (17)	0.37538 (12)	0.56996 (17)	0.0364 (4)	
N3	−0.10635 (16)	0.66905 (11)	0.35720 (17)	0.0430 (4)	
N4	−0.50763 (15)	0.45265 (15)	0.05912 (16)	0.0469 (4)	
C1	0.43749 (15)	0.47231 (17)	0.50384 (15)	0.0352 (3)	
C2	0.8180 (2)	0.36409 (15)	0.7887 (2)	0.0410 (5)	
C3	0.9495 (2)	0.43573 (13)	0.8856 (2)	0.0438 (5)	
C4	0.9417 (2)	0.56196 (15)	0.8789 (2)	0.0461 (5)	
C5	0.8050 (2)	0.62249 (14)	0.7738 (2)	0.0417 (5)	
C6	0.6711 (2)	0.55050 (13)	0.67435 (19)	0.0343 (5)	
C7	0.6765 (2)	0.42432 (13)	0.6819 (2)	0.0336 (4)	
C8	0.08069 (19)	0.50405 (15)	0.47829 (19)	0.0446 (5)	
C9	−0.09062 (19)	0.54887 (13)	0.36425 (19)	0.0371 (4)	
C10	−0.21640 (16)	0.46761 (16)	0.27169 (16)	0.0369 (3)	
C11	−0.36204 (19)	0.52325 (14)	0.1662 (2)	0.0383 (4)	
C12	−0.3806 (2)	0.64707 (13)	0.1538 (2)	0.0434 (5)	
C13	−0.2491 (2)	0.71654 (15)	0.2537 (2)	0.0454 (5)	
C14	−0.1877 (2)	0.33262 (15)	0.2842 (2)	0.0542 (6)	
H1	0.494 (2)	0.6529 (16)	0.516 (2)	0.055 (5)*	
H2	0.82390	0.27830	0.79360	0.0480*	
H3	1.04750	0.39780	0.95950	0.0500*	
H4	1.03430	0.60730	0.94910	0.0530*	
H5	0.80050	0.70840	0.76880	0.0490*	
H12	−0.48000	0.68360	0.08020	0.0510*	
H13	−0.26100	0.80210	0.24930	0.0540*	
H81	0.05890	0.43470	0.53870	0.0520*	
H82	0.13040	0.56640	0.55450	0.0530*	
H141	−0.29960	0.29300	0.26470	0.0660*	
H142	−0.12570	0.30650	0.20380	0.0660*	
H143	−0.12000	0.31340	0.39320	0.0660*	

### Atomic displacement parameters (Å^2^)

**Table:**

	*U*^11^	*U*^22^	*U*^33^	*U*^12^	*U*^13^	*U*^23^
S1	0.0266 (1)	0.0382 (2)	0.0387 (2)	0.0014 (2)	0.0019 (1)	−0.0015 (2)
O1	0.0386 (5)	0.0509 (6)	0.0422 (6)	0.0037 (4)	0.0051 (4)	0.0085 (4)
O2	0.0521 (7)	0.0478 (7)	0.0793 (9)	−0.0140 (6)	0.0116 (6)	−0.0158 (6)
O3	0.0467 (6)	0.1104 (13)	0.0441 (6)	−0.0083 (7)	−0.0019 (5)	−0.0079 (7)
N1	0.0350 (7)	0.0330 (6)	0.0412 (7)	0.0040 (5)	0.0029 (6)	0.0029 (6)
N2	0.0302 (6)	0.0355 (6)	0.0401 (7)	−0.0010 (5)	0.0015 (5)	0.0009 (6)
N3	0.0369 (7)	0.0406 (7)	0.0516 (8)	−0.0051 (5)	0.0110 (6)	−0.0030 (5)
N4	0.0320 (6)	0.0581 (9)	0.0502 (7)	−0.0062 (7)	0.0088 (5)	−0.0179 (8)
C1	0.0258 (5)	0.0395 (7)	0.0381 (6)	−0.0008 (7)	0.0030 (4)	−0.0012 (8)
C2	0.0389 (8)	0.0372 (8)	0.0435 (8)	0.0058 (7)	0.0028 (7)	0.0037 (7)
C3	0.0322 (7)	0.0528 (10)	0.0399 (8)	0.0058 (6)	−0.0043 (6)	0.0029 (7)
C4	0.0360 (8)	0.0552 (9)	0.0414 (9)	−0.0054 (7)	−0.0019 (7)	−0.0057 (8)
C5	0.0401 (8)	0.0343 (7)	0.0480 (9)	−0.0037 (6)	0.0053 (7)	−0.0025 (7)
C6	0.0311 (8)	0.0373 (8)	0.0350 (8)	0.0025 (6)	0.0089 (6)	0.0032 (6)
C7	0.0226 (7)	0.0407 (8)	0.0355 (8)	−0.0005 (5)	0.0032 (6)	0.0015 (5)
C8	0.0312 (7)	0.0580 (10)	0.0420 (8)	0.0063 (6)	0.0038 (6)	0.0046 (6)
C9	0.0280 (7)	0.0433 (8)	0.0399 (8)	0.0042 (5)	0.0079 (6)	0.0028 (6)
C10	0.0304 (5)	0.0385 (6)	0.0433 (6)	0.0012 (8)	0.0116 (5)	0.0006 (9)
C11	0.0290 (7)	0.0430 (8)	0.0449 (8)	−0.0024 (5)	0.0126 (7)	−0.0055 (6)
C12	0.0366 (8)	0.0427 (8)	0.0490 (9)	0.0051 (6)	0.0066 (6)	0.0053 (7)
C13	0.0431 (9)	0.0355 (7)	0.0566 (9)	0.0007 (6)	0.0097 (7)	0.0000 (7)
C14	0.0431 (9)	0.0394 (8)	0.0812 (13)	0.0029 (7)	0.0172 (9)	0.0010 (8)

### Geometric parameters (Å, °)

**Table:**

S1—O1	1.4831 (12)	C6—C7	1.396 (2)
S1—C1	1.7806 (13)	C8—C9	1.515 (2)
S1—C8	1.8460 (16)	C9—C10	1.409 (2)
O2—N4	1.224 (2)	C10—C11	1.393 (2)
O3—N4	1.2229 (19)	C10—C14	1.508 (2)
N1—C1	1.363 (2)	C11—C12	1.377 (2)
N1—C6	1.381 (2)	C12—C13	1.380 (2)
N2—C1	1.317 (2)	C2—H2	0.9500
N2—C7	1.413 (2)	C3—H3	0.9500
N3—C9	1.3336 (19)	C4—H4	0.9500
N3—C13	1.333 (2)	C5—H5	0.9500
N4—C11	1.478 (2)	C8—H81	0.9500
N1—H1	0.881 (17)	C8—H82	0.9500
C2—C3	1.384 (2)	C12—H12	0.9500
C2—C7	1.400 (2)	C13—H13	0.9500
C3—C4	1.397 (2)	C14—H141	0.9500
C4—C5	1.372 (2)	C14—H142	0.9500
C5—C6	1.406 (2)	C14—H143	0.9500
			
O1—S1—C1	104.84 (7)	C9—C10—C14	121.46 (13)
O1—S1—C8	106.76 (7)	N4—C11—C12	115.39 (14)
C1—S1—C8	98.26 (6)	N4—C11—C10	121.95 (14)
C1—N1—C6	105.78 (12)	C10—C11—C12	122.67 (15)
C1—N2—C7	103.05 (12)	C11—C12—C13	117.33 (15)
C9—N3—C13	118.24 (14)	N3—C13—C12	123.01 (15)
O2—N4—O3	124.37 (15)	C3—C2—H2	122.00
O2—N4—C11	118.23 (13)	C7—C2—H2	122.00
O3—N4—C11	117.38 (15)	C2—C3—H3	119.00
C1—N1—H1	129.6 (11)	C4—C3—H3	119.00
C6—N1—H1	123.1 (11)	C3—C4—H4	119.00
N1—C1—N2	115.09 (12)	C5—C4—H4	119.00
S1—C1—N1	121.48 (13)	C4—C5—H5	122.00
S1—C1—N2	123.35 (13)	C6—C5—H5	122.00
C3—C2—C7	116.71 (14)	S1—C8—H81	110.00
C2—C3—C4	121.98 (15)	S1—C8—H82	110.00
C3—C4—C5	122.11 (15)	C9—C8—H81	110.00
C4—C5—C6	116.34 (14)	C9—C8—H82	109.00
C5—C6—C7	121.95 (14)	H81—C8—H82	109.00
N1—C6—C5	131.98 (14)	C11—C12—H12	122.00
N1—C6—C7	106.07 (13)	C13—C12—H12	121.00
C2—C7—C6	120.90 (14)	N3—C13—H13	118.00
N2—C7—C6	110.00 (13)	C12—C13—H13	119.00
N2—C7—C2	129.10 (14)	C10—C14—H141	109.00
S1—C8—C9	107.75 (10)	C10—C14—H142	110.00
N3—C9—C8	114.20 (13)	C10—C14—H143	109.00
N3—C9—C10	124.54 (14)	H141—C14—H142	109.00
C8—C9—C10	121.22 (13)	H141—C14—H143	109.00
C9—C10—C11	114.19 (15)	H142—C14—H143	109.00
C11—C10—C14	124.31 (14)		
			
O1—S1—C1—N1	31.07 (13)	C3—C2—C7—C6	0.4 (2)
O1—S1—C1—N2	−145.56 (12)	C2—C3—C4—C5	−1.2 (3)
C8—S1—C1—N1	−78.80 (12)	C3—C4—C5—C6	0.9 (2)
C8—S1—C1—N2	104.57 (12)	C4—C5—C6—N1	179.22 (16)
O1—S1—C8—C9	51.34 (12)	C4—C5—C6—C7	0.0 (2)
C1—S1—C8—C9	159.64 (11)	N1—C6—C7—N2	0.34 (18)
C6—N1—C1—S1	−177.91 (10)	N1—C6—C7—C2	179.94 (14)
C6—N1—C1—N2	−1.02 (17)	C5—C6—C7—N2	179.71 (14)
C1—N1—C6—C5	−178.93 (16)	C5—C6—C7—C2	−0.7 (2)
C1—N1—C6—C7	0.35 (17)	S1—C8—C9—N3	−99.34 (14)
C7—N2—C1—S1	178.01 (10)	S1—C8—C9—C10	78.74 (15)
C7—N2—C1—N1	1.18 (16)	N3—C9—C10—C11	1.2 (2)
C1—N2—C7—C2	179.54 (16)	N3—C9—C10—C14	178.94 (14)
C1—N2—C7—C6	−0.90 (17)	C8—C9—C10—C11	−176.73 (13)
C13—N3—C9—C8	176.84 (14)	C8—C9—C10—C14	1.1 (2)
C13—N3—C9—C10	−1.2 (2)	C9—C10—C11—N4	179.93 (14)
C9—N3—C13—C12	−0.1 (2)	C9—C10—C11—C12	0.1 (2)
O2—N4—C11—C10	36.3 (2)	C14—C10—C11—N4	2.2 (2)
O2—N4—C11—C12	−143.88 (15)	C14—C10—C11—C12	−177.62 (15)
O3—N4—C11—C10	−145.52 (15)	N4—C11—C12—C13	178.95 (14)
O3—N4—C11—C12	34.3 (2)	C10—C11—C12—C13	−1.2 (2)
C7—C2—C3—C4	0.5 (2)	C11—C12—C13—N3	1.2 (2)
C3—C2—C7—N2	179.93 (17)		

### Hydrogen-bond geometry (Å, °)

**Table:**

*D*—H···*A*	*D*—H	H···*A*	*D*···*A*	*D*—H···*A*
N1—H1···N2^i^	0.881 (17)	2.553 (18)	3.425 (2)	170.5 (13)
C2—H2···O1^ii^	0.95	2.33	3.251 (2)	164.
C12—H12···O2^iii^	0.95	2.55	3.164 (2)	122.

Symmetry codes: (i) −*x*+1, *y*+1/2, −*z*+1; (ii) −*x*+1, *y*−1/2, −*z*+1; (iii) −*x*−1, *y*+1/2, −*z*.
